# P129, a pyrazole ring-containing isolongifolanone-derivate: synthesis and investigation of anti-glioma action mechanism

**DOI:** 10.1007/s12672-024-00858-9

**Published:** 2024-01-06

**Authors:** Yining Jiang, Yunyun Wang, Liyan Zhao, Wenzhuo Yang, Lin Pan, Yang Bai, Yubo Wang, Yunqian Li

**Affiliations:** 1 Department of Neurosurgery, First Hospital of Jilin University, No.71, Xinmin Street, Changchun, 130021 Jilin People’s Republic of China; 2 School of Pharmacy and Jiangsu Province Key Laboratory for Inflammation and Molecular Drug Target, Nantong University, Nantong, 226001 China; 3 Department of Blood Transfusion, Second Hospital of Jilin University, Changchun, 130041 China; 4 Department of Neurosurgery, Cancer Hospital of Sun Yat Sen University, Guangzhou, 510060 China

**Keywords:** Apoptosis, CDK-2, Cell cycle arrest, Glioblastoma, isolongifolanone derivate, Pyrazole, Treatment

## Abstract

**Background:**

Cyclin-dependent kinase-2 (CDK-2) is an important regulatory factor in the G_1_/S phase transition. CDK-2 targeting has been shown to suppress the viability of multiple cancers. However, the exploration and application of a CDK-2 inhibitor in the treatment of glioblastoma are sparse.

**Methods:**

We synthesized **P129** based on isolongifolanone, a natural product with anti-tumor activity. Network pharmacology analysis was conducted to predict the structural stability, affinity, and pharmacological and toxicological properties of **P129**. Binding analysis and CETSA verified the ability of P129 to target CDK-2. The effect of **P129** on the biological behavior of glioma cells was analyzed by the cell counting kit-8, colony formation, flow cytometry, and other experiments. Western blotting was used to detect the expression changes of proteins involved in the cell cycle, cell apoptosis, and epithelial–mesenchymal transition.

**Results:**

Bioinformatics analysis and CETSA showed that **P129** exhibited good intestinal absorption and blood–brain barrier penetrability together with high stability and affinity with CDK-2, with no developmental toxicity. The viability, proliferation, and migration of human glioma cells were significantly inhibited by **P129** in a dose- and time-dependent manner. Flow cytometry and western blotting analyses showed G_0_/G_1_ arrest and lower CDK-2 expression in cells treated with **P129** than in the controls. The apoptotic ratio of glioma cells increased significantly with increasing concentrations of **P129** combined with karyopyknosis and karyorrhexis. Apoptosis occurred via the mitochondrial pathway.

**Conclusion:**

The pyrazole ring-containing isolongifolanone derivate **P129** exhibited promising anti-glioma activity by targeting CDK-2 and promoting apoptosis, indicating its potential importance as a new chemotherapeutic option for glioma.

**Supplementary Information:**

The online version contains supplementary material available at 10.1007/s12672-024-00858-9.

## Introduction

Glioma originates from glial cells and is one of the most common malignancies of the central nervous system, with an annual incidence of 3–6.4 cases per 100,000 people [1]. According to a 2021 classification, there are four grades of gliomas; grades III and IV classified as high-grade gliomas [2]. Glioblastoma (GBM) is the most common diffuse glioma, and the 5-year survival rate of patients with grade IV disease is less than 10% [3]. Aggressive surgery, followed by radiotherapy plus chemotherapy, is the current standard care for patients with GBM [4]. Although this treatment prolongs survival, it cannot prevent the recurrence of GBM because of its infiltrative growth, temozolomide resistance, and other characteristics [4, 5]. Although the appearance of tumor-treating fields can improve overall survival [3], this method has not yet brought widespread benefits to the patient population owing to its high price and long treatment time. Hence, the further exploration of reliable treatments is critical.

Cyclin-dependent kinase (CDK)-2 is serine/threonine kinase composed of 298 amino acids with a highly conserved protein structure containing a double-flap fold [6]. The abnormal expression or function of CDK-2 has been shown to be related to a variety of cancers, including glioma [7–9]. Isolongifolanone is a sustainable natural product produced by the oxidation of isolongifolene, which is usually extracted from turpentine oil [10]. Although isolongifolanone is primarily employed as a natural fragrance, it has been found to exhibit antioxidant, anti-inflammatory, anticancer, and neuroprotective effects [11]. Pyrazole is a classical five-membered heteroaromatic ring with diverse pharmacological effects, including anticancer, anti-inflammatory, anti-obesity, and other properties, which lay the foundation for the development of new pharmacological agents and lead compounds [12]. Among them, pyrazole-based anticancer agents have been widely used in clinical treatment [12]. Hence, building a functional pyrazole ring-containing isolongifolanone derivative has high potential, and our group has been working on the development and biological activities of turpentine and its derivatives [10, 13, 14]. In this study, we synthesized **P129** as a derivative of isolongifolanone and investigated its anti-glioma effects and the underlying mechanisms.

## Materials and methods

### Reagents

All chemical reagents used in the synthesis of **P129** were purchased from Adamas (Shanghai, China). Reactions were monitored via thin-layer chromatography on silica gel-precoated F254 plates (Qingdao Haiyang Chemical Co., Ltd., China). All products were purified using silica gel column chromatography (200–300 mesh, Qingdao Haiyang Chemical Co., Ltd., China). High-resolution mass spectrometry (Agilent Q-TOF mass spectrometer) and nuclear magnetic resonance (NMR, ^1^H NMR, and ^13^C NMR, Bruker AVANCE instrument, 600 MHz) were used to confirm the structure of compounds. Tetramethyl silane in CDCl_3_ or dimethyl sulfoxide (DMSO)‑*d6* was used as the internal standard for NMR spectra. Penicillin/streptomycin and RNase were provided by Cell Signaling Technology (Beverly, MA, USA). Cell counting kit-8 (CCK-8), propidium iodide (PI), protein phosphatase inhibitor, RIPA lysate, and electrochemiluminescence kit were purchased from Sigma (St. Louis, MO, USA). DMSO (Selleck Chemicals, Houston, TX, USA) was stored at – 20 ℃. The Annexin V-FITC apoptosis detection kit and JC-1 mitochondrial membrane potential (MMP) assay kit were purchased from Beyotime Biotechnology (Shanghai, China). Primary antibodies for the detection of Bcl-2, caspase 3, CDK-2, cleaved caspase 3, cleaved PARP-1, cyclin E2, P21^CIP1^, PARP-1, retinoblastoma (RB) protein, and secondary detection antibodies (goat anti-mouse IgG and goat anti-rabbit IgG) antibodies were purchased from Abcam (Cambridge, MA, USA). β-actin was obtained from Abmart (Shanghai, China); Bax, caspase 9, cleaved caspase 9, phosphorylated-RB (p-RB) from Cell Signaling Technology; and E2F transcription factor-1 (E2F1) from ProteinTech (Chicago, IL, USA). Dulbecco’s modified eagle medium (DMEM), minimum essential medium (MEM), and the BCA protein assay kit were purchased from Thermo Fisher Scientific (Waltham, MA, USA).

### Synthesis route of P129

**1,1,5,5-tetramethyl-8-oxooctahydro-2H-2,4a-methanonaphthalene-7-carbaldehyde** (compound** 1**): EtOH (5 mL) and 30 mL of THF were added in a 100-mL flask. NaOEt (1.78 *g*, 26.1 mmol, 1 equiv) was added, the flask was cooled to 0 ℃, and isolongifolanone (5.74 *g*, 26.1 mmol, 1 equiv) was added. After 10 min, ethyl formate (2.03 *g*, 27.4 mmol, 1.05 equiv) was added dropwise, the ice bath was removed, and the mixture was stirred overnight at room temperature. After the reaction, the mixture was evaporated and the residue was dissolved in EtOAc (20 mL). The solution was washed with brine, dried, and concentrated. The residue was used for the next reaction without further purification to produce compound** 1**.

**1,1,5,5-tetramethyl-8-oxooctahydro-2H-2,4a-methanonaphthalene-7-carbonitrile** (compound **2**): Hydroxylamine hydrochloride (1.5 equiv) was added to a solution of compound **1** (4.96 *g*, 20 mmol) in EtOH, and the solution was stirred at 80 ℃ overnight. Then, the reaction mixture was evaporated and the residue was dissolved in EtOAc (20 mL). The solution was washed with brine, followed by drying and concentration. Then, the residue was dissolved in MeOH-H_2_O solution, followed by MeONa (1.5 equiv) addition, and the mixture was stirred at room temperature for 1 h. The solid was collected by filtration and washed with water to obtain a solid white powder compound **2**.

**5,5,9,9-tetramethyl-2,4,5,6,7,8,9,9a-octahydro-5a,8-methanobenzo[g]indazol-3-amine** (compound **3**): EtOH (20 mL) was added to a solution of compound **2** (4.9 *g*, 20 mmol) in hydrazine monohydrochloride (1.44 *g*, 21 mmol), and the solution was stirred at 80 ℃ overnight. Then, the reaction mixture was evaporated and the residue was dissolved in EtOAc (20 mL). The solution was washed with saturated NaHCO_3_ solution (20 mL), followed by brine (20 mL), and then dried and concentrated. The residue was purified via silica gel column chromatography (DCM/MeOH = 40/1) to produce compound **3** as a yellow solid.

**Fig. 1:**
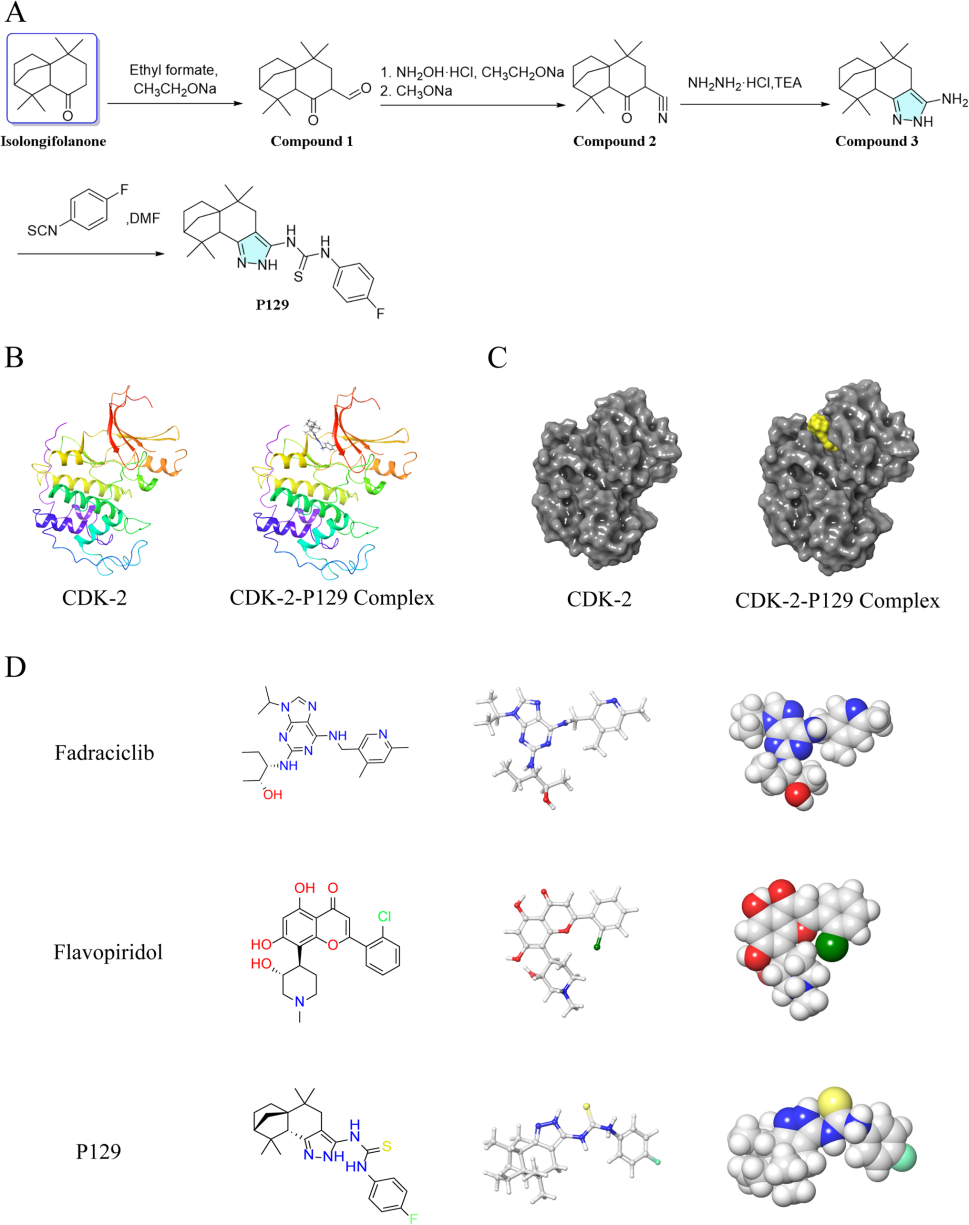
**A** Route of **P129** synthesis. **B** The initial molecular structure of CDK-2 and CDK-2–**P129** complex. **C** The surface of the complex is shown in yellow for **P129** and gray for CDK-2. **D** Schrödinger 2D and 3D structures of **P129**, fadraciclib, and fadraciclib

**1-(4-fluorophenyl)-3-(5,5,9,9-tetramethyl-2,4,5,6,7,8,9,9a-octahydro-5a,8-methanobenzo[g]indazol-3-yl)-thiourea** (**P129**). 4-Fluorophenyl isothiocyanate (1.0 equiv.) was added to a solution of compound **3** (0.259 g, 1 mmol) in DMF (10 mL), and the solution was stirred at room temperature overnight. After the reaction, the mixture was dissolved in EtOAc (20 mL). The solution was washed with brine, dried, and concentrated. Then, the residue was purified via silica gel column chromatography (EtOAc:PE = 1:6) to produce **P129** as a white solid at a yield of 60% (Fig. 1A). ^1^H NMR (400 MHz, DMSO-*d6*) *δ*: 12.21 (s, 1H), 11.80 (s, 1H), 10.14 (s, 1H), 7.58 (dd, *J* = 9.0, 5.0 Hz, 2H), 7.19 (t, *J* = 8.8 Hz, 2H), 2.39 (d, *J* = 15.6 Hz, 1H), 2.20 (d, *J* = 14.7 Hz, 2H), 1.80 (t, *J* = 10.6 Hz, 1H), 1.68 (d, *J* = 4.5 Hz, 2H), 1.51–1.39 (m, 2H), 1.23 (s, 3H), 1.17 (d, *J* = 9.7 Hz, 2H), 1.02 (s, 3H), 0.74 (s, 3H), 0.67 (s, 3H) (Supplementary Fig. 1).

### Prediction of pharmacological and toxicological properties based on structure using absorption, distribution, metabolism, and excretion (ADME) and TOPKAT

The Discovery Studio 2019 software (DS 2019; BIOVIA, San Diego, California, USA) provides researchers with easy-to-use tools for protein simulation, modification, and precision medicin [15, 16]. The 1.8-Å crystal structure of CDK-2 was downloaded from the Protein Database Bank (https://www.rcsb.org, Protein Data Bank identifier: 3PXY). The molecular structure of CDK-2 and the CDK-2/**P129** complex are shown in Fig. 1B, C. Fadraciclib (CYC065) is a second-generation aminopurine CDK-2/9 inhibitor with increased potency and selectivity toward CDK-2 and CDK-9 [17]. Flavopiridol, which is a novel flavone that competes with the ATP substrate to inhibit multiple CDKs, including CDK-1, CDK-2, and CDK-4 [18], was the first potent CDK inhibitor to be evaluated in clinical trials [18]. Deschloro-flavopiridol has been shown to co-crystallize with CDK-2 [19]. In this study, we selected fadraciclib and flavopiridol as the reference drugs. The 2D and 3D structures of CDK-2, fadraciclib, and flavopiridol are shown in Fig. 1D. The ADME levels of **P129**, fadraciclib, and flavopiridol, including water solubility, blood–brain barrier (BBB) permeability, cytochrome P4502D6 (CYP2D6) levels, liver toxicity, human intestinal absorption, and plasma protein levels were determined using the ADME module of DS 2019. The TOPKAT module was used to analyze the toxicological characteristics of the compounds, including rodent carcinogenicity, Ames mutagenicity, and developmental toxicity potential. The pharmacophores of the selected compounds and Lynparza were analyzed using the 3D-QSAR module of DS 2019. Only those with energies below 10 kcal/mol could be retained, and a maximum of 255 confirmations could be generated per molecule.

### Molecular docking simulation and pharmacological analysis

Studies on the molecular docking of ligands and proteins were conducted using the CDOCKER module of DS 2019. Using the CHARMm force field, CDOCKER is a method that generates accurate molecular docking statistics [20]. The CDOCKER algorithm is based on a simulated annealing protocol in which the receptor remains rigid and the ligand is allowed to bend and dock with protein residues within the binding site to identify the optimal binding mode. The section within a 5-Å radius of the ligand’s geometric center is referred to as the binding site spot. In addition, the best binding pose of the compounds to the protein was demonstrated using the Schrödinger software [21].

### Molecular dynamic simulation

The optimal binding conformation of the ligand–CDK-2 complex obtained from the previous molecular docking step was selected for molecular dynamic simulations. Using an explicit periodic boundary solvated water model, the ligand–receptor complex was contained in an orthogonal box and solvated. Sodium chloride was added to the solution with an ionic strength of 0.145 to mimic the physiological environment. The following simulation protocols were used for the system: 500 steps of steepest descent and conjugate gradient minimization; 5-ps equilibration simulations in a normal pressure ensemble at a temperature of 300 K (slowly driven from an initial temperature of 50 K); and 120-ps MD simulation (production module) at normal pressure and temperature with a time step of 1 fs. Long-range electrostatic calculations were performed using the particle mesh Ewald technique, and all hydrogen-containing bonds were fixed using an adaptation of the linear constraint solver technique. The DS 2019 analysis trajectory procedure was used to construct a trajectory for root-mean-square deviation (RMSD), potential energy, and structural parameters using the original complex configuration as a reference [21].

### Cell culture

Human LN18 and A172 glioblastoma cells were purchased from Shanghai Institutes for Biological Sciences (Shanghai, China). Human T98G, LN229 glioblastoma cells and human umbilical vein endothelial cells (HUVECs) were obtained from the American Type Culture Collection (Manassas, USA). Human LN18, A172, and LN229 were cultured in DMEM added with fetal bovine serum (10%) and 100 U/mL of penicillin–streptomycin. Human T98G cells were cultured in MEM added with fetal bovine serum (10%), penicillin–streptomycin (100 U/mL), and sodium pyruvate (100 U/mL). All cells were cultured in a humidified atmosphere of 5% CO_2_ at 37 ℃. HUVECs were cultured in a specific medium according to the provided instructions.

### Cellular thermal shift assay (CETSA)

A172 cells were collected and lysed using RIPA buffer containing protease inhibitors and phosphorylase inhibitors, followed by protein extraction on ice for 1 h. The cell lysates were divided into three aliquots and incubated with DMSO, 50 μM **P129**, and 100 μM **P129** for 1 h, respectively. Then, the lysates were heated at the indicated temperatures for 10 min (40–70 ℃) to denature proteins and quickly cooled on ice. Subsequently, the samples were centrifuged at 14,000 × *g* for 30 min at 4 ℃ to remove aggregates. The resulting supernatant was boiled with loading buffer for western blotting.

### Cell viability analysis

LN18, T98G, A172, LN229, and HUVECs were seeded in 96-well microplates with 200 μL of culture medium and incubated overnight. Thereafter, cells in the logarithmic growth phase were cultured for 24 h (3.0 × 10^3^ cells/well), 48 h (2.0 × 10^3^ cells/well), 72 h (1.5 × 10^3^ cells/well), and 96 h (1.0 × 10^3^ cells/well) in the presence of **P129** diluted in DMSO at 0, 5, 10, 20, 40, and 80 μM. Cell viability was assessed using the CCK-8 method and expressed as the ratio of the absorbance value at 570 nm of the control cells.

### Colony formation assay

LN18 and A172 (1 × 10^3^ cells/well) were seeded in 6-well microplates and incubated overnight. Then, the cells were treated with **P129** (0, 7.5, 15, and 30 μM) for approximately 10 days. Thereafter, the cells were fixed with 4% paraformaldehyde solution for 30 min and then stained with 0.1% crystal violet for 20 min. The number of colonies containing ≥ 50 cells in each well was counted under a microscope at 4 × magnification using ImageJ (NIH, Bethesda, MD, USA).

### Cell cycle analysis

LN18 (1.5 × 10^5^ cells/well) and A172 (2 × 10^5^ cells/well) in the logarithmic growth phase were transferred into 6-well microplates and cultured for 12 h. Thereafter, both cell lines were treated with **P129** (0, 7.5, 15, and 30 μM) for 48 h and then fixed with 70% pre-cooled ethanol overnight at – 20 ℃. Subsequently, the cells were collected and washed (× 2) with phosphate-buffered saline (PBS) and then incubated with PI/RNase dye in the dark for 20 min. The data for the stained cells were acquired using FACSCalibur flow cytometer (Becton Dickinson, San Diego, CA, USA) and analyzed using ModFit Version 4.1.7 (Verity Software House, USA).

### Apoptosis assay

Cellular apoptosis was assessed with the Annexin V/PI apoptosis detection kit according to the manufacturer’s instructions. LN18 and A172 (2.0 × 10^5^ cells/well) were seeded in 6-well microplates and cultured in the presence of **P129** (0, 7.5, 15, and 30 μM) for 48 h. The proportion of viable and apoptotic cells was then determined using the flow cytometric analysis of ≥ 10,000 cells, and the rate of cell apoptosis was calculated using ModFit Version 4.1.7 software.

### Hoechst 33342 staining

The morphological changes of glioma cells after **P129** treatment were evaluated by Hoechst 33342 staining. Briefly, LN18 (1.5 × 10^5^ cells/well) and A172 (2 × 10^5^ cells/well) were seeded in 6-well plates and then treated with **P129** for 48 h. Subsequently, the cells were washed with PBS and incubated with Hoechst 33342 stain in the dark for 15 min at 37 ℃. Finally, the cells were observed and randomly photographed using a fluorescence microscope (Olympus IX71, Tokyo, Japan).

### Measurement of MMP

MMP was detected using the JC-1 kit according to the manufacturer’s instructions. When the MMP is high, the JC-1 polymer aggregates in the matrix, emitting red fluorescence; when it is low, the monomer cannot gather in the matrix and emits green fluorescence. LN18 and A172 were seeded onto 6-well microplates at a density of 1.5 × 10^5^ cells/well overnight, followed by **P129** (0, 7.5, 15, and 30 μM) addition and culture for 48 h. Subsequently, the cells were stained with JC-1, incubated for 20 min, and washed with JC-1 buffer twice to remove excess stain. The change in the red/green fluorescence density ratio was monitored via fluorescence microscopy and flow cytometry, which reflected the condition of the mitochondria.

### Wound-healing assay

LN18 (5 × 10^5^ cells/well) and A172 (4 × 10^5^ cells/well) were seeded in 6-well microplates and cultured overnight. When the cells reached 90% confluence, a vertical scratch was introduced into the cell monolayer using a 1-mL micropipette tip, with the tip moved vertically downward to ensure a consistent wide scratch. Subsequently, the microplates were washed (× 3) with PBS and cultured in serum-free DMEM containing **P129** (0, 15, and 30 μM). A microscope equipped with a camera was used to monitor the migration of cells into the scratched areas at 0 h and 48 h.

### Cell migration (Transwell) assay

Matrigel-coated Transwell chambers (24-well; 8-μm pore size, Corning, New York, NY, USA) were used to evaluate the migration of glioma cells according to the manufacturer’s protocol. Suspensions of LN18 and A172 cells (2 × 10^4^) in 200 μL of serum-free medium were seeded into the upper chamber in the presence of **P129** (0, 7.5, 15, and 30 μM) while DMEM containing serum was added to the lower chamber and incubated for 48 h. Thereafter, the migrated cells located on the lower surface of the membrane were fixed with 4% methanol and stained with 0.1% crystal violet. The invasive cells were then counted and photographed under an inverted microscope (Olympus IX71, Tokyo, Japan).

### Western blotting

LN18 (1.5 × 10^5^ cells/well) and A172 cells (2 × 10^5^ cells/well) in the logarithmic growth phase were seeded in 6-well microplates and treated with **P129** (0, 7.5, 15, and 30 μM) for 48 h. Subsequently, the cells were collected and lysed with RIPA buffer containing protease inhibitors and phosphorylase inhibitors before protein extraction on ice. The sample volume was measured and protein concentration was determined using a BCA protein assay kit. Equal amounts of total protein for each sample were separated using SDS-PAGE and transferred to polyvinylidene fluoride membranes (Merck Millipore, Burlington, MA, USA). Subsequently, the membranes were blocked with 5% dried skimmed milk powder in Tris-buffered saline at room temperature for 2 h and then incubated with the following primary antibodies for 14 h at 4 ℃: mouse anti-E2F1 (66515, 1:5000), rabbit anti-β-actin (TA7018S, 1:1000), anti-Bax (5023 T, 1:1000), anti-Bcl-2 (ab32124, 1:1000), anti-caspase 3 (ab32351, 1:5000), anti-caspase 9 (9502 T, 1:1000), anti-CDK-2 (ab32147, 1:5000), anti-cleaved caspase 3 (ab32042, 1:500), anti-cleaved caspase 9 (52873 T, 1:1000), anti-cleaved PARP-1 (ab32561, 1:1000), anti-cyclin E2 (ab40890, 1:5000), anti-p-RB (8516 T, 1:1000), anti-P21 (ab109520, 1:5000), anti-PARP-1 (ab32138, 1:10000), and anti-RB (ab181616, 1:2000). Next, the membranes were washed (× 3; 10 min/wash) with PBS and incubated with the corresponding horseradish peroxidase-conjugated secondary antibodies (1:10,000) for 90 min at room temperature. After the membranes were washed again (× 3; 10 min/wash) with PBS, the protein bands were detected using an electrochemiluminescence method and visualized with a gel imaging system (Sage Creation Science Co., Ltd, Beijing, China).

### Statistical analysis

Quantitative experimental data were obtained from at least three independent experiments and expressed as the mean ± standard deviation. Data were analyzed via independent-samples *t*-test or one-way analysis of variance using GraphPad Prism software (Version 9.4.1; GraphPad Software Inc., La Jolla, CA, USA). *P* < 0.05 was set as the threshold for statistical significance.

## Results

### Prediction of pharmacological and toxicological properties

The ADME and TOPKAT modules of DS 2019 were used to predict the pharmacological and toxicological properties of **P129**, with fadraciclib and flavopiridol as positive controls. **P129** had inferior aqueous solubility but fair BBB penetration, intestinal absorption, and plasma protein binding levels, with no CYP2D6 inhibition or hepatotoxicity, compared to fadraciclib and flavopiridol (Table 1). In addition, **P129** exhibited no Ames mutagenicity or developmental toxicity potential (Table 2). Furthermore, compared to the positive controls, **P129** exhibited better BBB penetrability, which might be conducive to better therapeutic efficacy. As shown in Fig. 1D, **P129**, fadraciclib, and flavopiridol have similar five- or six-membered annular structures, nitrogen species atoms, and phenyl moieties, suggesting that they may have similar inhibitory functions, which we verified in subsequent bioinformatics analysis and wet experiments.

**Table 1:** Adsorption, distribution, metabolism, and excretion properties of compounds

Molecule	Aqueous-Solubility Level^a^	BBB Level^b^	CYP2D6^c^	Hepatotoxicity^d^	Absorption Level^e^	PPB Level^f^
Fadraciclib	2	3	0	1	0	0
Flavopiridol	3	3	0	1	0	1
P129	1	1	0	1	0	0

^a^Aqueous solubility level: 0, extremely low; 1, very low, but possible; 2, low; 3, good

^b^Blood–brain barrier (BBB) level: 0, very high penetrant; 1, high; 2, medium; 3, low; 4, undefined

^c^Cytochrome P4502D6 (CYP2D6): 0, non-inhibitor; 1, inhibitor

^d^Hepatotoxicity: 0, nontoxic; 1, toxic

^e^Human intestinal absorption level: 0, good; 1, moderate; 2, poor; 3, very poor

^f^Plasma protein binding (PPB) level: 0, weak; 1, strong

**Table 2:** Toxicities of compounds

Molecule	U.S. National Toxicology Program (NTP)				Ames	DTP
	Mouse/Female	Mouse/Male	Rat/Female	Rat/Male		
Fadraciclib	1	1	0	0	0.011	1
Flavopiridol	0.001	1	0	0.474	1	1
P129	1	0	1	1	0	0

Ames < 0.3 (nonmutagen); > 0.8 (mutagen)

DTP (developmental toxicity potential) < 0.3 (nontoxic); > 0.8 (toxic)

NTP (U.S. National Toxicology Program) < 0.3 (noncarcinogen); > 0.8 (carcinogen)

### Ligand binding analysis of P129–CDK-2

**Fig. 2:**
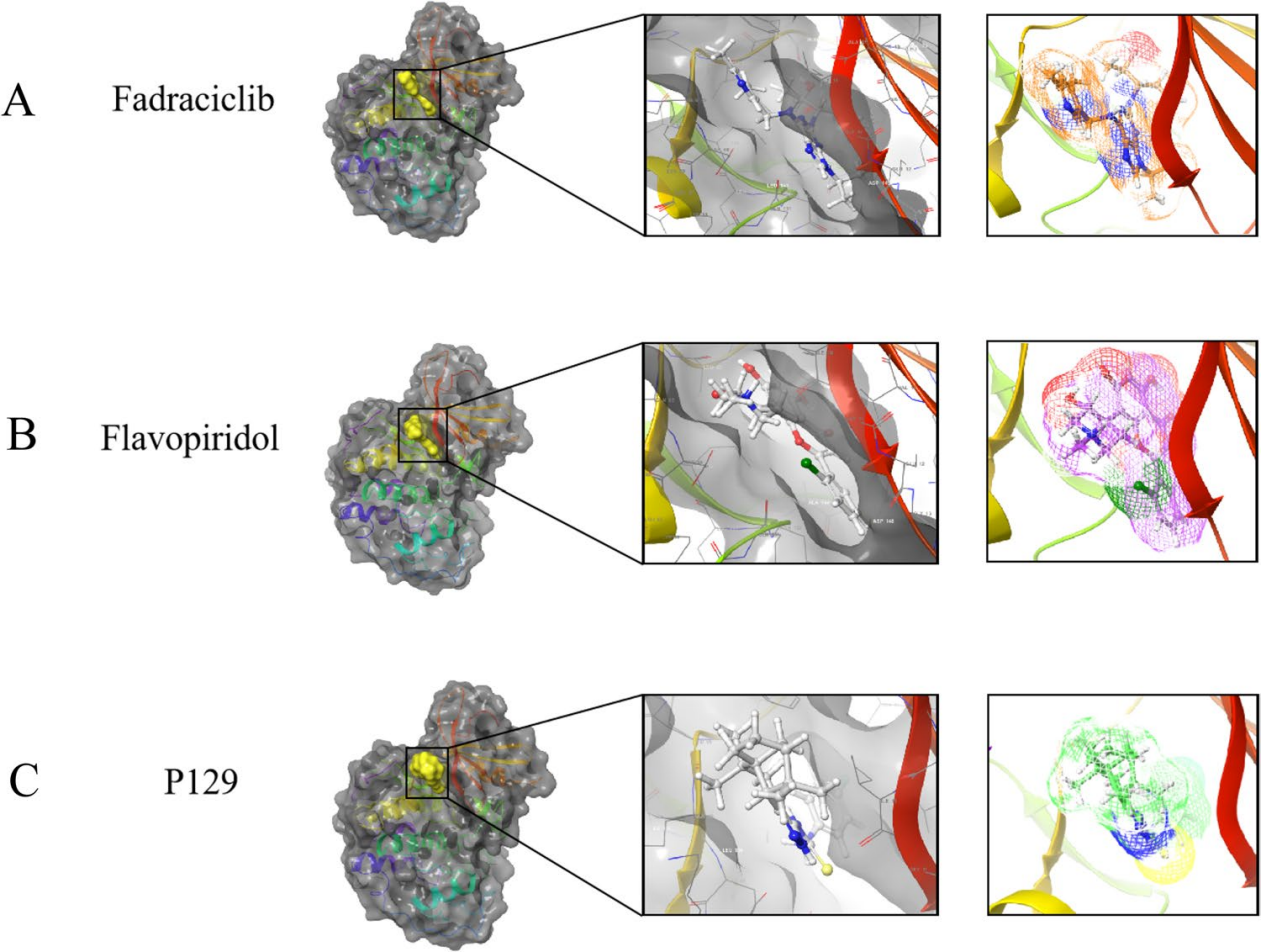
Schematic diagram of interactions between ligands and CDK-2 using Schrödinger. **A** Fadraciclib–CDK-2 complex. Structures and net electron cloud structures of fadraciclib are shown as orange sticks. **B** Flavopiridol–CDK-2 complex. Structures and net electron cloud structures of flavopiridol are shown as purple sticks. **C** **P129**–CDK-2 complex. Structures and net electron cloud structures of **P129** are shown as green sticks

**Fig. 3:**
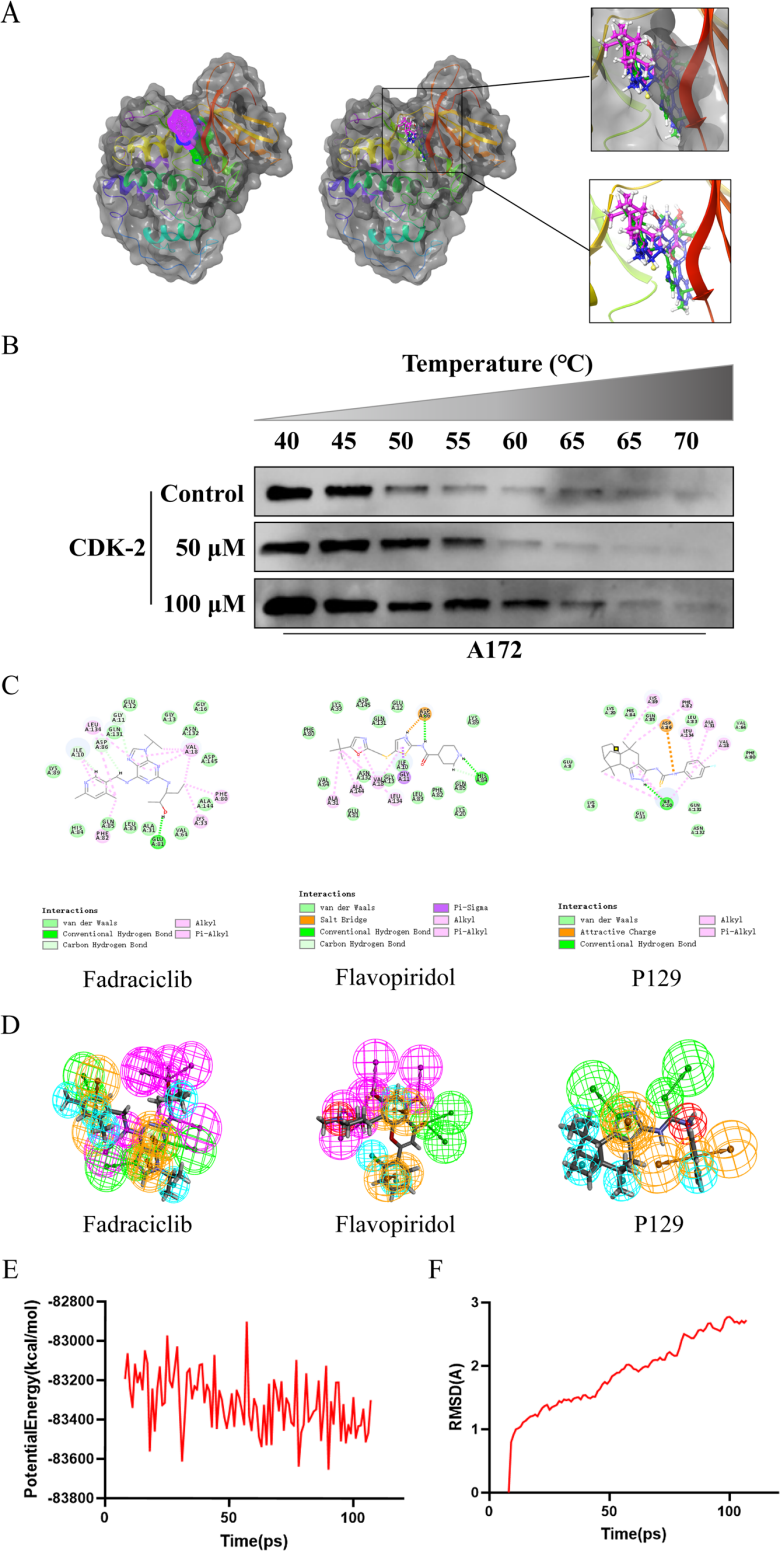
**A** A comparison of the spatial conformation of small molecules in the protein-binding pockets of the CDK-2 surface is shown in gray. Structures of fadraciclib, flavopiridol, and **P129** are shown in green, blue, and purple sticks, respectively. **B** CETSA revealed that because of binding with **P129**, the concentration of undegraded CDK-2 protein gradually increased along with the increasing concentration of **P129**. **C** Schematic diagram of intermolecular interactions of fadraciclib, flavopiridol, and **P129** with the binding pockets of CDK-2 predicted using DS 2019. **D** The 3D-QSAR module of DS 2019 used for pharmacophore prediction. Green represents hydrogen acceptors, blue represents hydrophobic centers, purple represents hydrogen donors, and yellow represents aromatic rings. **E** The potential energy estimated using the molecular dynamics simulation of the **P129**–CDK-2 complex. **F** Average backbone root-mean-square deviation (RMSD) changes in the **P129**–CDK-2 complex

**P129** was accurately connected to the binding pocket of CDK-2, and the mechanism of ligand binding was examined using the CDOCKER module. As shown in Table 3, the interaction energy of **P129** (-66.2813 kcal/mol) was lower than that of fadraciclib (− 61.4454 kcal/mol) and flavopiridol (− 52.1807 kcal/mol), indicating a higher binding affinity to CDK-2 than the positive controls. By applying DS 2019 and Schrödinger software, we performed a comprehensive analysis of the ligand conformation in the CDK-2 binding pocket and the protein–ligand complex interaction; the binding pattern of **P129** to the CDK-2 binding pocket is shown in Fig. 2. As shown in Fig. 3A, there was a significant overlap between **P129** and the two positive references in the binding pocket posture. **P129**, fadraciclib, and flavopiridol are essentially identical in the way they bind and interact with CDK-2, confirming the potential of **P129** to exert similar inhibitory effects on CDK-2. To further verify that CDK-2 is indeed the target of **P129**, CETSA was performed. As shown in Fig. 3B, as the temperature increased, CDK-2 was gradually degraded. However, **P129 binding** increased the concentration of undegraded CDK-2, and the trend further right-shifted as the concentration of **P129** increased. Through precise analysis using DS 2019, we obtained details of the interaction between ligand and protein, including bond length, bond type, and bond atoms (Fig. 3C, Supplementary Table 1). The results showed that **P129** forms one pair of conventional hydrogen bonds with CDK-2 (**P129**:H56- A:ILE10:O) and nine pairs of hydrophobic bonds (A:ILE10-**P129**, **P129**:C11-A:ILE10, **P129**:C13-A:LYS89, A:PHE82-**P129**:C14, **P129**-A:ILE10, **P129**-A:ILE10, **P129**-A:VAL18, **P129**-A:ALA31, **P129**-A:LEU134). Among them, the conventional hydrogen bond length formed by **P129** and CDK-2 are smaller than those of flavopiridol, indicating a relatively strong hydrogen bond. These results indicated that **P129** might bind to CDK-2 with higher affinity than fadraciclib and flavopiridol, suggesting broad application prospects for **P129**.

**Table 3:** CDOCKER potential energy of compounds with CDK-2

Molecule	-CDOCKER Interaction energy (Kcal/mol)
Fadraciclib	− 61.4454
Flavopiridol	− 52.1807
P129	− 66.2813

### Pharmacophore analysis and molecular dynamic simulation of P129–CDK-2

According to the evaluation of the feature pharmacophores using the 3D-QSAR module of DS 2019, **P129** has 11 characteristic pharmacophores, including 4 hydrophobic centers, 3 hydrogen bond acceptors, 1 ionizable positive, and 4 aromatic rings, whereas fadraciclib has 25, and flavopiridol has 20 (Fig. 3D). The characteristic pharmacophores of **P129** are basically similar to those in fadraciclib and flavopiridol (Table 4). Molecular dynamics simulations were performed in simulated natural environments to evaluate the stability of the **P129**–CDK-2 complex. The potential energy of the compound became stable over time and the trajectory of the complex basically reached equilibrium after 100 ps (Fig. 3E). In addition, the RMSD of **P129** increased gradually after 100 ps (Fig. 3F). Molecular dynamics simulation showed that the interaction between **P129** and CDK-2 is beneficial to the stability of the complex. These data indicated that the **P129**–CDK-2 complex could exist stably in the natural environment and inhibit CDK-2 activity.

**Table 4:** Analysis of feature pharmacophores

Molecule	Total	HB_acceptor	HB_donor	Hydrophobic	Positive_ionizable	Ring_aromatic
Fadraciclib	24	7	6	5	0	6
Flavopiridol	20	7	5	3	1	4
P129	11	2	0	4	1	4

*HB* hydrogen bond

### P129 inhibits the proliferation of glioma cells

**Fig. 4:**
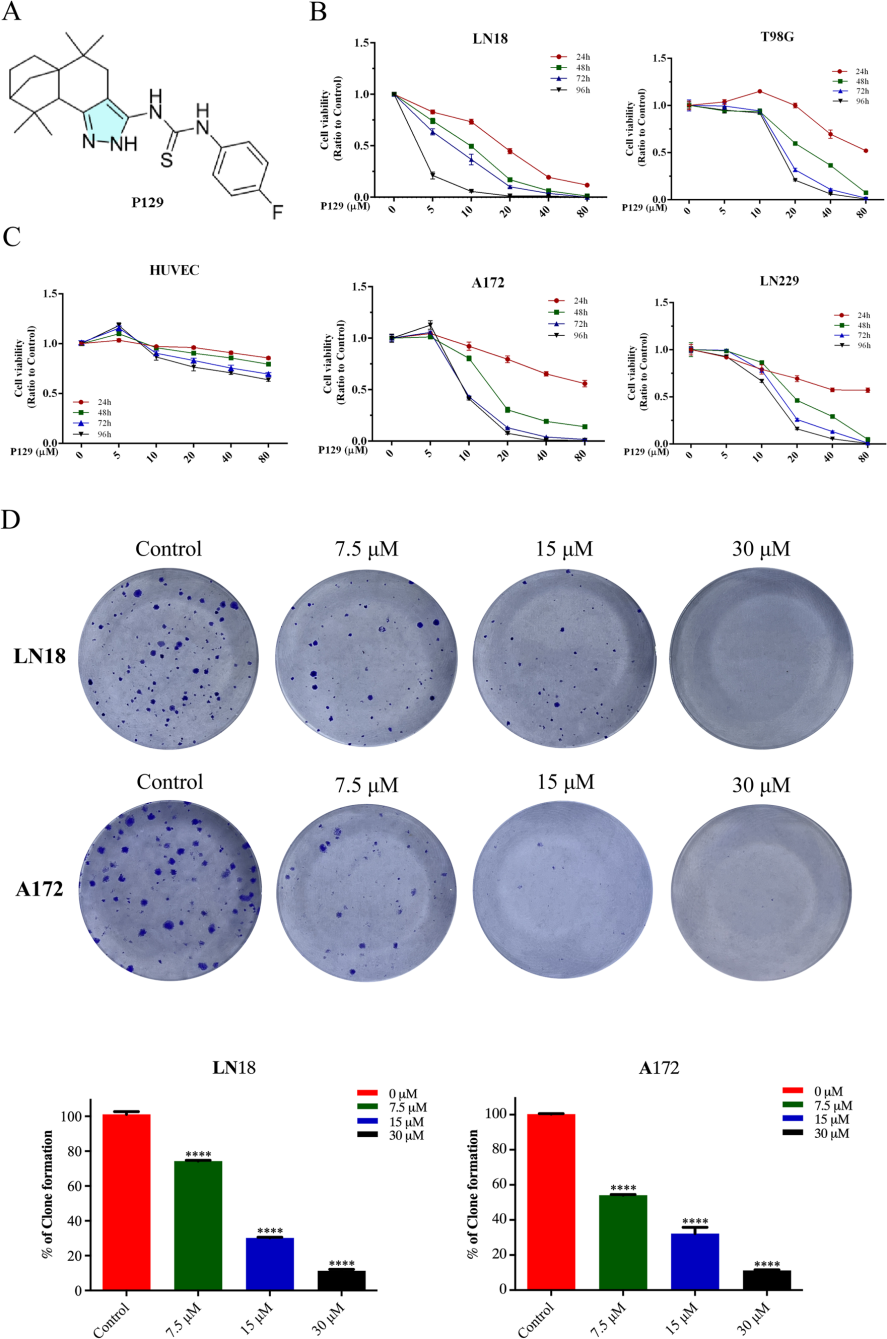
**P129** inhibited the viability and proliferation of glioma cells. **A** Chemical structure of **P129**. **B** CCK-8 assay showed that the growth of human LN18, T98G, A172, and LN229 glioma cells was inhibited by **P129** in a dose- and time-dependent manner. **C** **P129** toxicity was significantly limited in HUVECs compared with GBM cells. **D** Colony quantitation and representative images of colonies formed by LN18 and A172 glioma cells after 10 days of treatment with **P129** at the indicated concentrations. ****p < 0.0001 compared to the control group

To explore the toxicity of **P129** (Fig. 4A) on glioma cells, we measured the viability of LN18, T98G, A172, and LN229 cells treated with **P129** using CCK-8 assays. The viability of glioma cells decreased in a time- and dose-dependent manner as the concentration of **P129** increased (Fig. 4B). The 50% inhibiting concentrations (IC_50_) of **P129** in LN18, T98G, A172, and LN229 cells were 9.532 μM, 30.097 μM, 13.374 μM, and 19.875 μM, respectively, after 48-h treatment. Among them, the IC_50_ of LN18 and A172 was the lowest, suggesting that these two cell lines were the most sensitive to **P129** treatment compared with T98G and LN229. Therefore, LN18 and A172 were selected for subsequent experiments. The IC_50_ of LN18 cells at 24 h, 48 h, 72 h, and 96 h was 17.016 μM, 9.532 μM, 7.213 μM, and 2.781 μM and that of A172 cells was 21.462 μM, 13.374 μM, 9.737 μM, and 9.671 μM, respectively. The evaluation of the toxicity of **P129** to HUVECs as normal human cells revealed a slight reduction in viability, indicating the limited toxic effects of **P129** on normal cells (Fig. 4C). Interestingly, at low concentrations, **P129** promoted the proliferation of T98G, A172, and HUVECs, whereas high concentrations inhibited proliferation. Colony formation assays were performed to further explore the long-term inhibitory effects of **P129**. The results showed that the number of glioma cell colonies decreased significantly as the concentration of **P129** increased (Fig. 4D), further suggesting that **P129** could inhibit the long-term viability of glioma cells.

### P129 induces G_1_ phase arrest in glioma cells

**Fig. 5:**
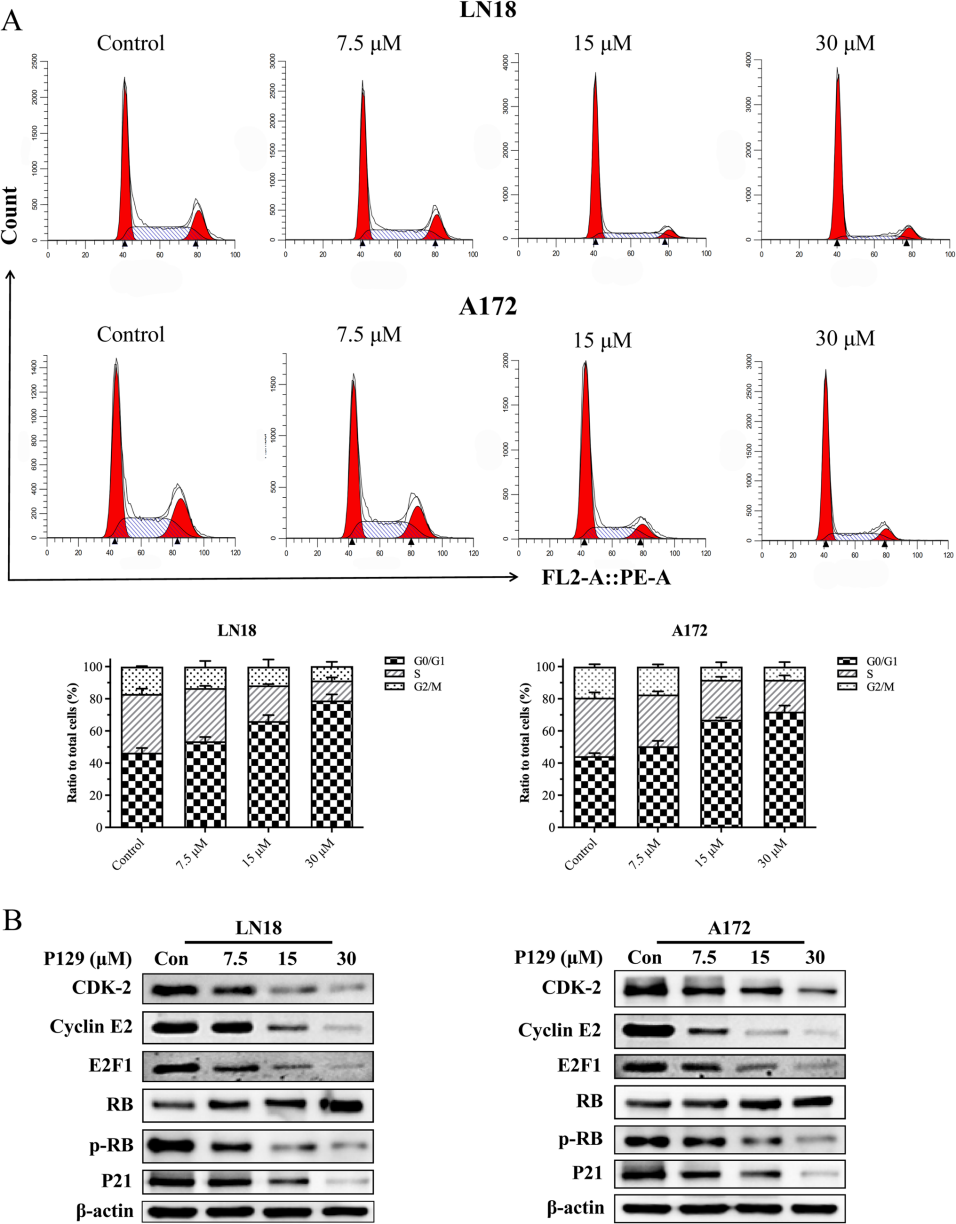
**P129** induced G_0_/G_1_ phase cell cycle arrest in glioma cell lines. **A** Flow cytometry analysis of the cell cycle phase distribution of **P129**-treated LN18 and A172 cells. Histograms showing the percentage of LN18 and A172 cells in the G_0_/G_1_, S, and G_2_/M phases. **B** Western blot analysis of CDK-2, Cyclin E2, E2F1, RB, p-RB, and P21 levels in LN18 and A172 cells treated with **P129** for 48 h. β-actin was used as the loading control

**P129** was synthesized to target CDK-2, which plays a key role in regulating the cell cycle; therefore, we further investigated the number of LN18 and A172 cells in each phase as well as the expression level of CDK-2 and its downstream changes. The results showed a significant and dose-dependent increase in the number of cells in the G_0_/G_1_ phase (Fig. 5A), indicating that **P129** induced G_1_-phase arrest in glioma cells. To explore the inhibitory effects of **P129** on CDK-2, the expression level of CDK-2 and other proteins involved in G_1_ phase regulation was determined via the western blot analysis of LN18 and A172 cells after treatment with **P129** for 48 h. The results showed a dose-dependent decrease in the expression of CDK-2, cyclin E2, E2F1, and p-RB, and the CIP/KIP family member p21^KIP1^ (Fig. 5B). These results provided evidence that **P129** effectively inhibited its target CDK-2 and confirmed the failure of transition from the G_1_ phase to the S phase.

### P129 induces cell apoptosis in GBM

**Fig. 6:**
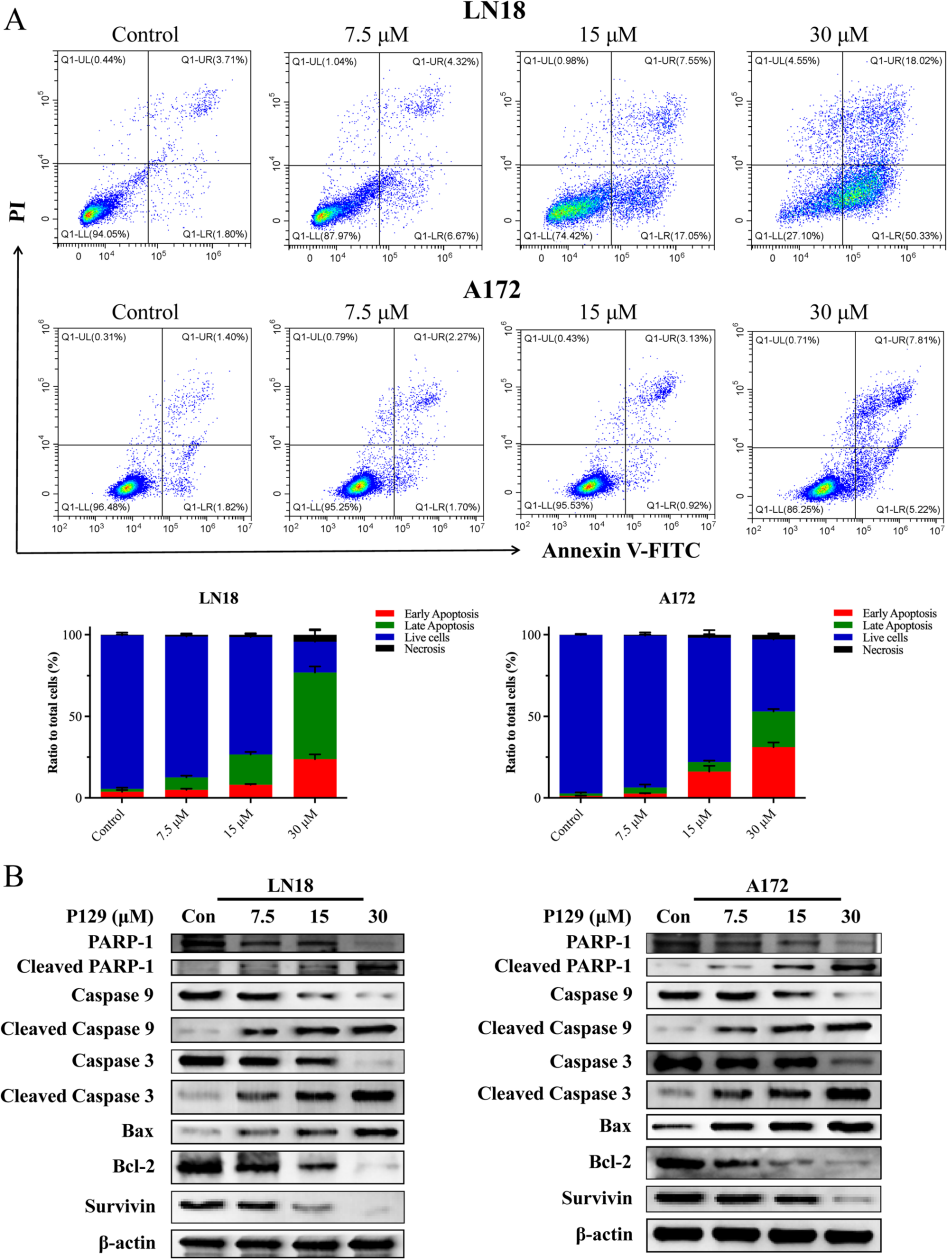
**P129** induced a caspase-dependent mitochondrial apoptosis of glioma cells. **A** Representative flow cytometry images showing the expression levels of Annexin V and PI double-labeled LN18 and A172 glioma cells after **P129** treatment. Histograms showing the percentage of LN18 and A172 early apoptotic, late apoptotic, live, and necrotic cells. **B** Western blot analysis of the expression levels of PARP-1, cleaved PARP-1, caspase-9, cleaved caspase-9, caspase-3, cleaved caspase-3, Bax, Bcl-2, and survivin after **P129** treatment

**Fig. 7:**
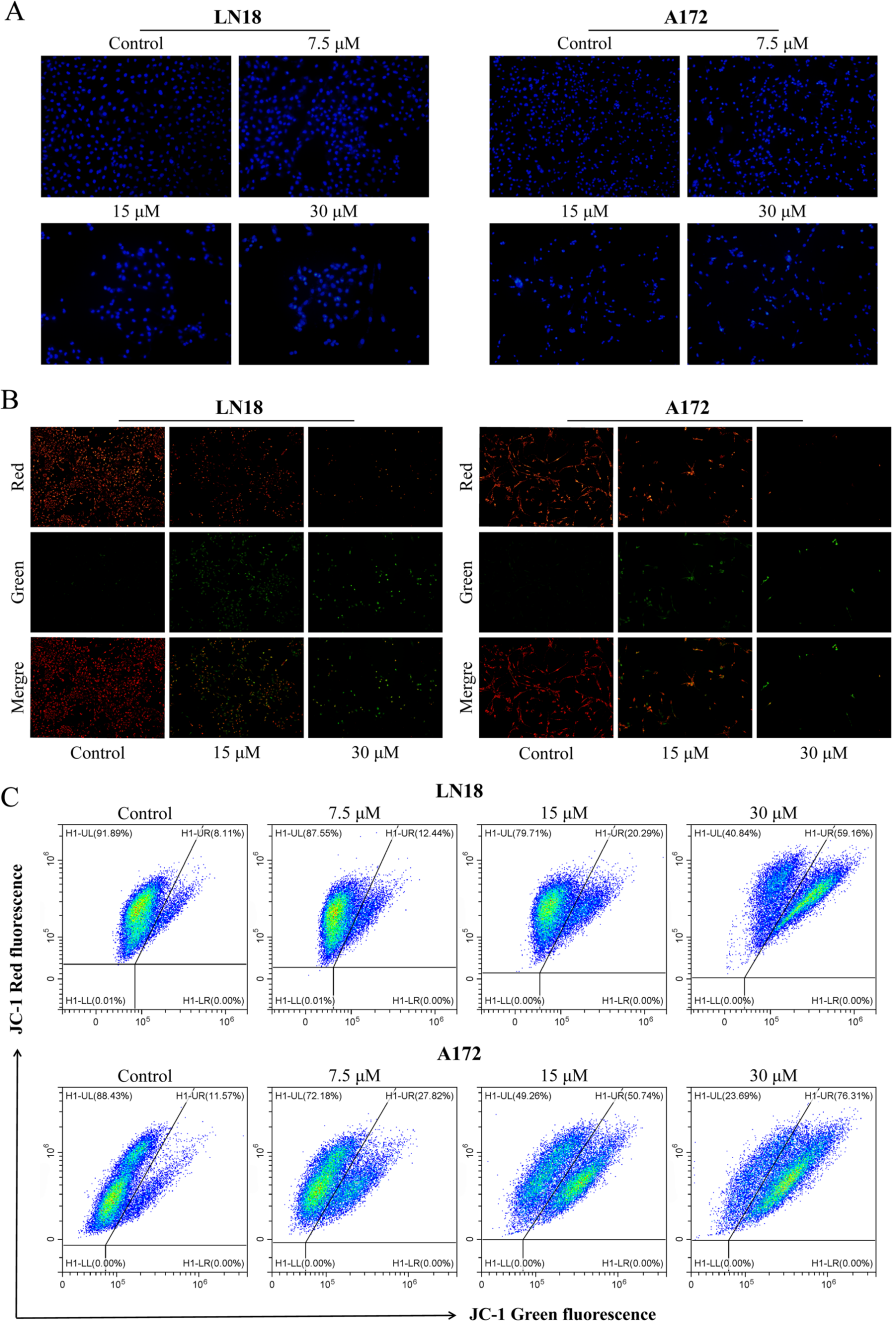
**A** Representative fluorescence and bright-field images showing the morphological changes in LN18 and A172 cells treated with **P129** for 48 h and stained with Hoechst 33342 (20 × magnification). **B** Representative fluorescence images of LN18 and A172 with JC-1 staining after treatment with **P129** for 48 h. **C** MMP detection via the JC-1 mitochondrial fluorescent probe in LN18 and A172 cells after **P129** treatment for 48 h analyzed using flow cytometry

To investigate whether **P129** inhibited the proliferation of glioma cells by inducing apoptosis, LN18 and A172 were treated with **P129** for 48 h and the number of apoptotic cells was evaluated via the flow cytometric analysis of Annexin V/PI staining. The results showed that the proportion of PI-labeled early apoptotic cells increased after treatment in a dose-dependent manner (Fig. 6A). To further confirm these results, Hoechst 33,342, a sensitive nuclear stain for living cells, was used to detect morphologic changes in LN18 and A172. As shown in Fig. 7A, the density of heavily blue-stained apoptotic cells increased with the increasing concentrations of **P129**. To further explore the mechanism of apoptosis, we detected the expression levels of related endogenous apoptosis proteins. Western blotting analysis revealed a concentration-dependent increase in the expression levels of cleaved PARP-1, cleaved caspase 3, and cleaved caspase 9, accompanied by the decreased expression of PARP-1, caspase 3, and caspase 9 (Fig. 6B). The increased expression of cleaved PARP-1 further confirmed the activation of caspase 3. In addition, the western blot analysis of two mitochondrial-related apoptosis regulators, the pro-apoptotic protein Bax and the anti-apoptotic protein Bcl-2, showed a significant increase in Bax expression, whereas Bcl-2 and survivin levels decreased in a dose-dependent manner after treatment with **P129** for 48 h (Fig. 6B). Moreover, MMP detection with JC-1 showed a **P129**-dose-dependent decrease in red fluorescence and green fluorescence enhancement via fluorescence microscopy (Fig. 7B) and flow cytometry (Fig. 7C). Thus, our findings confirmed that **P129** efficiently induced the caspase-dependent mitochondrial apoptosis of glioma cells.

### P129 suppresses the migration and invasion of glioma cells by inhibiting epithelial–mesenchymal transition (EMT)

**Fig. 8:**
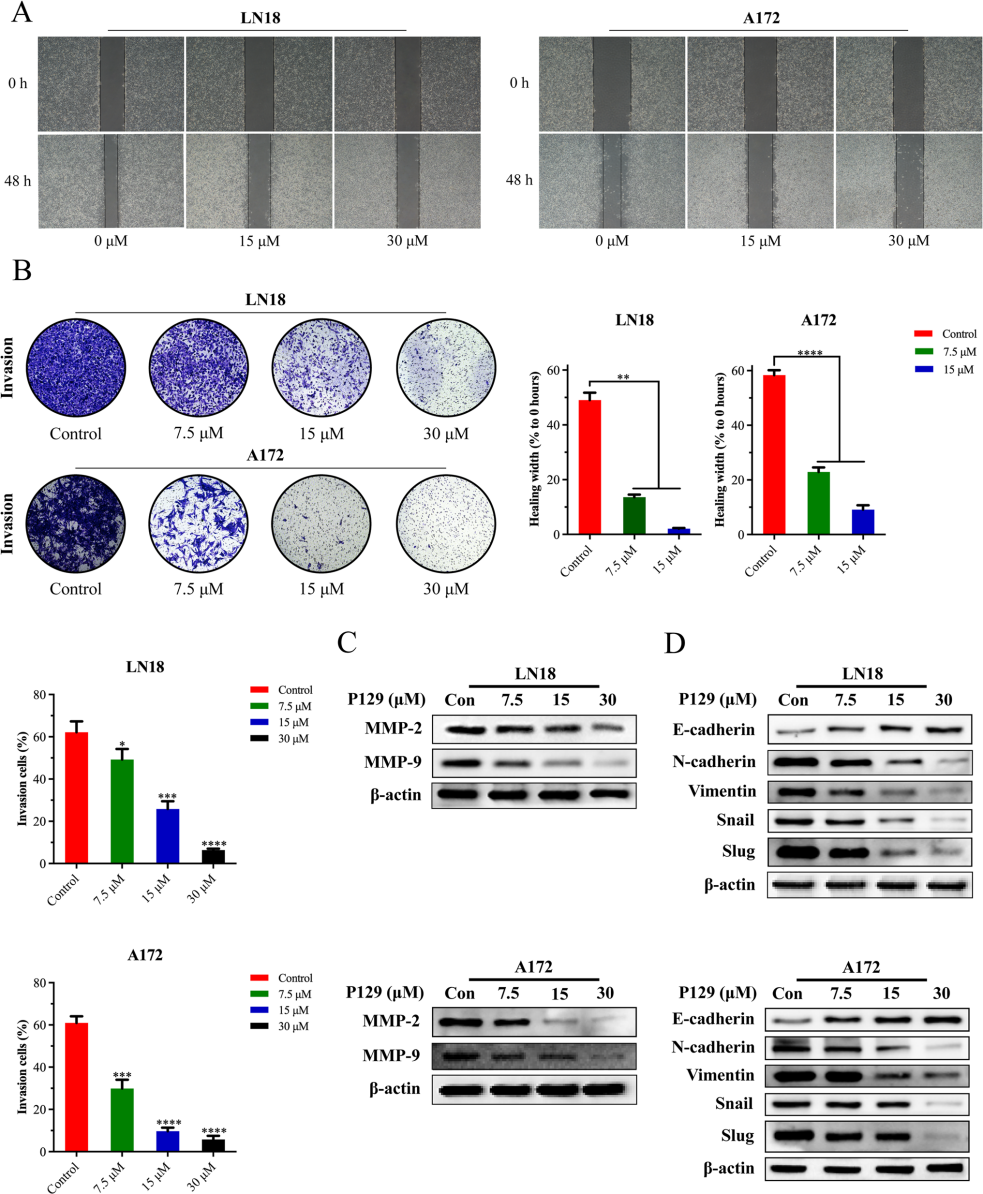
**P129** inhibited the migration and invasion of glioma cells. **A** **P129** suppressed the migration ability of LN18 and A172 cells in wound-healing assays. **B** Matrigel-coated Transwell invasion assays showing the inhibitory effect of **P129** on the invasive ability of LN18 and A172 cells. **C** Western blot analysis showing the expression level of MMP-2 and MMP-9 proteins in LN18 and A172 cells following treatment with **P129**. **D** Western blot analysis of the expression level of EMT-related proteins (E-cadherin, N-cadherin, vimentin, Snail, Slug). Representative images and histograms illustrating the migration and invasion capacity of glioblastoma cells. *p < 0.05, **p < 0.01, ***p < 0.001, ****p < 0.0001 compared to the control group

Having provided evidence that **P129** inhibited the viability of glioma cells, we further investigated its ability to inhibit metastasis. Wound-healing assays suggested that **P129** induced a significant and dose-dependent decrease in the migration ability of LN18 and A172 glioma cells (Fig. 8A). Similar results were obtained using Transwell assays that reflect the invasion ability of glioma cells (Fig. 8B). Moreover, we then explored the inhibitory mechanism of **P129**. Western blot analysis showed that the expression levels of MMP-2 and MMP-9 proteins decreased in a dose-dependent manner after treatment with **P129** for 48 h (Fig. 8C). The additional analysis of EMT-related proteins indicated that **P129** inhibited the invasive ability of glioma cells by blocking EMT. Specifically, after treatment with **P129** for 48 h, the expression of N-cadherin, vimentin, Snail, and Slug was reduced significantly, whereas the expression of E-cadherin was increased in a dose-dependent manner (Fig. 8D). Overall, these data provide evidence that **P129** could inhibit glioma cell metastasis via the downregulation of MMP-2 and MMP-9 and inhibition of EMT.

## Discussion

Glioma is the most common malignant primary tumor of the central nervous system, with high rates of morbidity and mortality [2]. Among them, GBM is the deadliest glioma, accounting for 70% of all diffuse gliomas, with a median overall survival time of 15 months [22]. In addition to its rapid proliferation, extensive invasion, chemotherapy resistance, and genetic heterogeneity, the lack of timely diagnosis and sensitive therapeutic monitoring tools have always been the primary reasons for the poor prognosis of GBM [1, 23]. Hence, effective glioma therapy remains an unmet need. Thus, new therapies are urgently needed, and the development of chemotherapeutic drugs or chemopreventive agents based on natural products is a promising direction for these efforts. Turpentine is an abundant renewable resource with longifolene as its main component [10]. Isolongifolanone is obtained from the oxidation of longifolene and has some advantages in anticancer drug development, including fair cell membrane penetrability, low toxicity, high water solubility, and low cost [24]. These characters were further verified in our network pharmacological analysis.

In this study, **P129** was synthesized and its inhibitory effect on glioma was analyzed. First, the binding of **P129** and CDK-2 was verified via CDOCKER module analysis and CETSA. Subsequently, a series of network pharmacological analyses, including the modules of ADME, TOPKAT, CDOCKER, and molecular dynamic simulation, showed the promising pharmacological and toxicological characteristics, stability, and affinity of **P129**. The underlying molecular mechanisms and targeting of CDK-2 were further explored. We proved that **P129** could effectively inhibit the proliferation of GBM cells in a dose- and time-dependent manner, including LN18, T98G, A172, and LN229. Only mild toxicity was observed in normal cells. The sustained inhibitory effect was further verified using the colony formation assay. Different sensitivities to different glioma cell lines were found, with IC_50_ values ranging from 9.532 μM to 30.097 μM, which might be associated with different cell line origins and protein expression profiles [25]. Moreover, a slight low-concentration promotion of proliferation was observed in T98G, A172, and HUVECs, which is also a property of many chemotherapeutic drugs, such as cinobufagin [26]. Flow cytometric and immunoblotting analyses confirmed that **P129** blocked the cell cycle in the G_0_/G_1_ phase by targeting CDK-2 and that DNA damage subsequently induced mitochondria-mediated apoptosis. The western blotting assays showed dose-dependent increases in cleaved caspase 3, cleaved caspase 9, cleaved PARP-1, and Bax and decreases in Bcl-2. Caspase 9 has been considered a significant regulator of apoptosis via the mitochondrial pathway [27]. The depolarization of MMP and changes in Bcl-2 family proteins further confirmed this finding. Hoechst 33342 staining was used to observe morphological changes in glioma cells and showed obvious blue staining enhancement, nuclear fragmentation, and nuclear enrichment, which are the features of apoptosis. Therefore, **P129** could effectively inhibit the proliferation and migration of GBM cells and kill glioma cells by inhibiting the cell cycle in the G_0_/G_1_ phase and inducing intrinsic apoptosis. Moreover, a significant dose- and time-dependent inhibition of migration and invasion by **P129** was observed. Invasion is an important link in cancer progression, and matrix metalloproteinases are the family most closely associated with this process. Uncontrolled tumor progression, local invasion, and tumor metastasis are largely dependent on the proteolytic activity of matrix metalloproteinases, which inhibit immune cell recruitment and tissue integrity by releasing cell surface-bound cytokines and matrikines, degrading the extracellular matrix [28, 29]. MMP-2 and MMP-9 are extracellular proteolytic enzymes participating in the invasion and tumor progression of glioma, and our results showed significant **P129**-induced MMP-2 and MMP-9 inhibition. The EMT process involves the loss of intercellular adhesion and apicobasal polarity and the acquisition of mesenchymal features and motility. In cancer, the program is hijacked, and motility fuels invasion [30]. In this study, we found that N-cadherin, Snail, and Slug were decreased in LN18 and A172 cells after **P129** treatment. Summing up, **P129** could inhibit the invasion of glioma by suppressing MMP-2 and MMP-9 and EMT progression.

CDK-2 plays a critical role in the G_1_ and G_2_ phases of the cell cycle. In the absence of mitogens, CDK-2 is usually maintained in the inactive state by the upstream CDK inhibitors p21^CIP1^ and p27^KIP1^ of the CIP/KIP families. CDK2 is usually activated by forming complexes with cyclin E and cyclin A to promote mitosis [6]. Following its activation, the CDK-2–cyclin E complex phosphorylates RB, which drives the E2F-mediated transcription of cyclin E, cyclin D-CDK-4/6 mediated sequestration of p21^CIP1^ and p27^KIP1^, and ubiquitin-mediated proteolysis of p21^CIP1^ and p27^KIP1^ [6]. These steps lead to the increase in the complexes of CDK-2-cyclin E and CDK-2-cyclin A, which allow duplication initiation and S phase entry. In the S phase, CDK-2 is further phosphorylated, stimulating its kinase activity, and the complex CDK-2-cyclin A further phosphorylates RB to complete the most significant activity of the S phase-DNA replication [31]. Thus, CDK-2 plays a critical role in the cell cycle regulation of a series of biological processes, including DNA damage repair, intracellular transportation, signal transduction, and DNA and RNA translation. In this study, the compound **P129** was synthesized to target CDK-2, and the cell cycle analysis revealed a significant dose-dependent arrest of glioma cells in the G_1_, with the absence of S-phase cells. Thus, we concluded that **P129** treatment inhibited the transition of glioma cells from G_0_/G_1_ to S.

In eukaryotic cells, cell cycle progression usually depends on the replication of genomic DNA and subsequent segregation into daughter cells. However, there is compelling evidence that cancer cells are unable to exit the cell cycle leading to uncontrolled cell division [6, 32]. More importantly, most functional elements that control the cell cycle are also essential for cancer cell viability, which provides new opportunities for chemotherapy and immunotherapy targets. Although CDK-2 expression in glioma cells is not very high compared with other tumor types according to *the Human Protein Atlas* (www.proteinatlas.org, Version 22.0), CDK-2 and its cyclin partners play an important role in regulating the G_1_/S phase transition and are usually abnormally activated in glioma [33, 34], thereby implicating CDK-2 as a target for the design of therapeutic inhibitors for GBM. In accordance with this, the results of the current study further verified the therapeutic potential of inhibiting glioma cells. Overall, CDK-2 is involved in a variety of oncogenic signaling pathways owing to its importance in processes such as the cell cycle, apoptosis, and chemotherapy resistance. The CDK-4/6 inhibitor palbociclib was shown to inhibit CDK-2 indirectly by elevating the abundance of p21^CIP1^ [35]. The clinical exploration of CDK-4/6 inhibitors has led to the improved outcomes of some cancer treatments and an increased focus on the exploration of other CDKs. CDK-2 and cyclin E are fundamentally linked with some specific cancer types, including glioma and ovarian cancer, among others [35, 36]. Moreover, the cyclin E_1_ locus is frequently amplified in human malignancies, especially in ovarian cancer and breast cancer but also in glioma [37, 38]. The abovementioned evidence suggests that specific CDK-2 targeting inhibitors may be a feasible therapy in anti-glioma treatment. This study reported the effectiveness of **P129** in inhibiting GBM cell proliferation and promoting their apoptosis by CDK-2 targeting. GBM treatment has long depended on chemotherapy (such as temozolomide), but this approach lacks specificity and faces extensive drug resistance [39]. In the present study, although a novel glioma treatment based on natural product derivatives was proposed, its specific targeting mechanisms have not been effectively addressed. The decline of P21 at the protein level appears to suggest the same. Therefore, “specific targeting” research will remain our focus in the future.

CDK-2 is known to play an important role in cell apoptosis by regulating the functional components of the apoptotic pathways [35]. Forkhead box O1 (FOXO1), the downstream target of CDK-2, plays a critical role in the DNA damage induction aspect of early apoptosis. In the absence of CDK-2-mediated phosphorylation of the inhibitory site of FOXO1, the resulting DNA damage induces G1/S phase arrest and early apoptosis [40]. Moreover, FOXO1 inactivation can trigger exogenous apoptosis by upregulating pro-apoptotic proteins, including TRAIL, Bim, and FasL [40, 41]. Faber et al*.* also reported that the inhibition of CDK-2 by CVT-313 or CDK-2-siRNA induced early apoptosis and reduced the level of myeloid cell leukemia-1 in diffuse large B-cell lymphoma cells [42]. CDK-2 inhibition has been considered in the treatment of highly aneuploid cancers, especially in KRAS-mutant lung cancer [43]. CDK-2 inhibition led to anaphase apoptosis and further inhibited the growth of lung cancer xenografts [44]. The use of CDK-2 inhibitors has the potential to be extended for the treatment of other cancers because many cancers have aneuploid cells with supernumerary centrosomes, which is consistent with the results of glioma cell treatment with **P129** in this study. Hence, it may be feasible to increase the potency of CDK-2 inhibitors through combination with other treatments. Combining fadraciclib, the positive control in our study, with eribulin showed improved effects in triple-negative breast cancer [45]. Furthermore, fadraciclib combined with a PI3K inhibitor showed synergistic toxicity in serous uterine carcinoma with CCNE1 amplification [46]. Azimi et al*.* also reported that the combination of the selective CDK inhibitor dinaciclib with a heat shock protein 90 (Hsp90) inhibitor showed high effectiveness in melanoma cell lines with BRAF and Hsp90 inhibition [47]. Thus, the combination of **P129** with other treatment molecules will be further explored in follow-up work. In addition, CDK-2 affects hormone-dependent cancers by phosphorylating the receptors of androgen, estrogen, and progesterone and increasing their transcriptional activity and CDK-2 inhibition has been reported to prevent the progression of prostate cancer and breast cancer [48, 49]. A stem-like subpopulation of inflammatory breast cancer cells exhibits CDK-2–cycling E complex overexpression, which renders the cells chemotherapy-resistant. This resistance was rescued by the combination of paclitaxel with the CDK-2 inhibitor SU9516, which induced a significant increase in early apoptosis [50]. CDK-2 is the culmination of most resistance signals, including C-MYC, cyclin E, and cyclin D_1_ overexpression; RB inactivation, and p21^CIP1^ or p27^KIP1^ reduction [51, 52], which further illustrate the importance of designing mature and stable CDK-2 inhibitors.

CDK-2 overexpression is positively associated with recurrence risk, and higher expression of CDK-2 has been found in metastasis [35]. Similarly, according to the Chinese Glioma Genome Atlas (www.cgga.org.cn), CDK-2 expression is increased in IDH wildtype, high-grade, and recurrent glioma. The broad functionality of CDK-2 in proliferative and pro-survival pathways highlights its potential as an ideal target for mechanism-based and low-toxicity therapeutic strategies in cancer treatment.

## Conclusion

In this study, we designed and synthesized **P129**, an isolongifolanone derivate containing a pyrazole ring. The CDK-2-targeting and glioma-inhibiting effects of **P129** were confirmed using a series of network pharmacological predictions and in vitro experiments. This was the first application of isolongifolanone and its derivative in the treatment of glioma. The emergence of **P129** fills the gap in CDK-2 inhibitors for the treatment of glioma and offers new opportunities to improve the therapeutic outcomes of glioma patients. Thus, **P129** warrants further development by researchers from different specialties to achieve its translation into the clinic as an effective anti-glioma treatment.

### Supplementary Information

Additional file1 (DOCX 632 KB)

## Data Availability

The original contributions presented in the study are included in the article/supplementary material, and further inquiries can be directed to the corresponding author/s.

## Abbreviations

ADME: Absorption, distribution, metabolism and excretion
BBB: Blood–brain barrier
CCK-8: Cell counting kit-8
CDK: Cyclin-dependent kinase
CETSA: Cellular thermal shift assays
CYP2D6: Cytochrome P4502D6
DMEM: Dulbecco’s modified eagle medium
DMSO: Dimethyl sulfoxide
DS 2019: Discovery Studio 2019 software
E2F1: E2F transcription factor-1
EMT: Epithelial–mesenchymal transition
FOXO1: Forkhead box O1
GBM: Glioblastoma
Hsp90: Heat shock protein 90
HUVECs: Human umbilical vein endothelial cells
IC_50_: 50% Inhibiting concentration
MEM: Minimum essential medium
MMP: Mitochondrial membrane potential
NMR: Nuclear magnetic resonance
PBS: Phosphate-buffered saline
p-RB: Phosphorylated-retinoblastoma protein
RB: Retinoblastoma protein
RMSD: Root-mean-square deviation
WHO: World Health Organization
