# Supplementary material for: P129, a pyrazole ring-containing isolongifolanone-derivate: synthesis and investigation of anti-glioma action mechanism
Source: Discov Oncol. 2024 Jan 6;15:6. doi: 10.1007/s12672-024-00858-9 (PMC10771574; doi:10.1007/s12672-024-00858-9)
Supplement: Supplementary file 1 — Additional file1 (DOCX 632 KB) [file 12672_2024_858_MOESM1_ESM.docx]

*Supplementary Material*

**P129, a pyrazole ring-containing isolongifolanone-derivate: Synthesis and investigation of anti-glioma action mechanism**

**Yining Jiang^1^**^†^**, Yunyun Wang^2^**^†^**, Liyan Zhao^3^**^†^**, Wenzhuo Yang^4^**^†^**, Lin Pan^1^, Yang Bai^1^, Yubo Wang^1^, Yunqian Li^1*^**

***Corresponding Author:**

Dr. Yunqian Li;

Department of Neurosurgery, First Hospital of Jilin University;

71, Xinmin Street; Changchun 130021, Jilin, P.R. China.

Email: yunqian@jlu.edu.cn

# Supplementary Figures and Tables

**1.1 Supplementary Figure**

**Figure 1. Identification of P129, a pyrazole ring-containing isolongifolanone-derivate with NMR spectra.**

**2.2 Supplementary Table**

**Table 1: Hydrogen Bond and hydrophobic interaction parameters for each compound with CDK-2.**

| **Receptor** | **Molecule** | **Category** | **Types** | **Donor atom** | **Receptor atom** | **Distance(Å)** |
| --- | --- | --- | --- | --- | --- | --- |
| **3PXY** | **Fadraciclib** | Hydrogen Bond | Conventional Hydrogen Bond | Fadraciclib:H60 | A:GLU81:O | 1.94734 |
|  |  | Hydrogen Bond | Carbon Hydrogen Bond | A:GLY11:HA2 | Fadraciclib:N9 | 2.50041 |
|  |  | Hydrogen Bond | Carbon Hydrogen Bond | Fadraciclib:H44 | A:ASP86:OD2 | 2.53401 |
|  |  | Hydrogen Bond | Carbon Hydrogen Bond | Fadraciclib:H46 | A:ILE10:O | 2.76266 |
|  |  | Hydrophobic | Alkyl | Fadraciclib:C1 | A:VAL18 | 3.88598 |
|  |  | Hydrophobic | Alkyl | Fadraciclib:C1 | A:LYS33 | 4.46638 |
|  |  | Hydrophobic | Alkyl | Fadraciclib:C13 | A:VAL18 | 4.64502 |
|  |  | Hydrophobic | Alkyl | Fadraciclib:C25 | A:ILE10 | 5.01039 |
|  |  | Hydrophobic | Alkyl | Fadraciclib:C25 | A:LEU134 | 5.27936 |
|  |  | Hydrophobic | Pi-Alkyl | A:PHE80 | Fadraciclib:C1 | 4.27233 |
|  |  | Hydrophobic | Pi-Alkyl | A:PHE82 | Fadraciclib:C25 | 4.98328 |
|  |  | Hydrophobic | Pi-Alkyl | Fadraciclib | A:VAL18 | 4.10997 |
|  |  | Hydrophobic | Pi-Alkyl | Fadraciclib | A:LEU134 | 5.16568 |
|  |  | Hydrophobic | Pi-Alkyl | Fadraciclib | A:VAL18 | 4.64642 |
|  |  | Hydrophobic | Pi-Alkyl | Fadraciclib | A:ILE10 | 4.31082 |
|  | **Flavopiridol** | Hydrogen Bond | Conventional Hydrogen Bond | Flavopiridol:H46 | A:ILE10:O | 2.74148 |
|  |  | Hydrogen Bond | Conventional Hydrogen Bond | Flavopiridol:H47 | A:LEU83:O | 1.94082 |
|  |  | Hydrogen Bond | Conventional Hydrogen Bond | Flavopiridol:H49 | A:LEU83:O | 2.79902 |
|  |  | Hydrogen Bond | Carbon Hydrogen Bond | A:GLN85:HA | Flavopiridol:O27 | 2.75675 |
|  |  | Hydrogen Bond | Carbon Hydrogen Bond | Flavopiridol:H29 | A:ASP86:OD2 | 2.30607 |
|  |  | Hydrogen Bond | Carbon Hydrogen Bond | Flavopiridol:H30 | A:ILE10:O | 2.4415 |
|  |  | Hydrogen Bond | Carbon Hydrogen Bond | Flavopiridol:H32 | A:ASP86:OD2 | 2.31651 |
|  |  | Hydrogen Bond | Carbon Hydrogen Bond | Flavopiridol:H33 | A:ILE10:O | 2.22906 |
|  |  | Hydrogen Bond | Carbon Hydrogen Bond | Flavopiridol:H45 | A:ASP86:OD2 | 2.32898 |
|  |  | Hydrophobic | Pi-Sigma | A:VAL18:HG11 | Flavopiridol | 2.90561 |
|  |  | Hydrophobic | Pi-Alkyl | Flavopiridol | A:VAL18 | 5.17875 |
|  |  | Hydrophobic | Pi-Alkyl | Flavopiridol | A:ALA31 | 4.07607 |
|  |  | Hydrophobic | Pi-Alkyl | Flavopiridol | A:LEU134 | 4.15991 |
|  |  | Hydrophobic | Pi-Alkyl | Flavopiridol | A:ALA31 | 5.29815 |
|  |  | Hydrophobic | Pi-Alkyl | Flavopiridol | A:LEU134 | 4.63395 |
|  |  | Hydrophobic | Pi-Alkyl | Flavopiridol | A:ALA144 | 4.70052 |
|  |  | Hydrophobic | Pi-Alkyl | Flavopiridol | A:VAL18 | 5.30381 |
|  | **P129** | Hydrogen Bond | Conventional Hydrogen Bond | p129:H56 | A:ILE10:O | 2.39868 |
|  |  | Hydrophobic | Alkyl | A:ILE10 | p129 | 5.00941 |
|  |  | Hydrophobic | Alkyl | p129:C11 | A:ILE10 | 5.27963 |
|  |  | Hydrophobic | Alkyl | p129:C13 | A:LYS89 | 5.04578 |
|  |  | Hydrophobic | Pi-Alkyl | A:PHE82 | p129:C14 | 5.39134 |
|  |  | Hydrophobic | Pi-Alkyl | p129 | A:ILE10 | 4.5748 |
|  |  | Hydrophobic | Pi-Alkyl | p129 | A:ILE10 | 5.24789 |
|  |  | Hydrophobic | Pi-Alkyl | p129 | A:VAL18 | 4.6768 |
|  |  | Hydrophobic | Pi-Alkyl | p129 | A:ALA31 | 4.73134 |
|  |  | Hydrophobic | Pi-Alkyl | p129 | A:LEU134 | 4.31236 |
